# Russell Bodies and Russell Body Inflammatory Polyp in the Colorectum: A Review of Clinicopathologic Features

**DOI:** 10.1155/2018/2845291

**Published:** 2018-07-29

**Authors:** Heidi Reinhard, Dipti M. Karamchandani

**Affiliations:** Department of Pathology, Division of Anatomic Pathology, Penn State Milton S. Hershey Medical Center and Penn State College of Medicine, Hershey, PA 17033, USA

## Abstract

Colorectal mucosa with Russell bodies is a reactive inflammatory lesion composed of mature plasma cells, known as Mott cells which contain multiple intracytoplasmic eosinophilic globules. To the best of our knowledge, 3 case reports of colorectal Russell body containing lesions have been reported in the English literature (searched from 1980 to date), including just one case report of Colonic Russell body inflammatory polyp. Their importance lies in being aware of this unusual entity, recognizing it as well as the clinical scenario in which this typically arises and differentiating it from its malignant mimics that come in the histologic differential. This review discusses the clinical and endoscopic presentation, histopathologic features, ancillary studies, pathogenesis, differential diagnosis, prognosis, and treatment of this rare lesion.

## 1. Introduction

Russell bodies are eosinophilic spherical or globular cytoplasmic inclusions that accumulate in the rough endoplasmic reticulum of mature plasma cells. These plasma cells containing Russell bodies are also known as Mott cells [1]. Mott cells were first described in 1890 by William Russell [2]; however the first case of Russell bodies in the gastrointestinal tract was in the stomach and was not described until 1998 by Tazawa and Tsutsumi [3]. Colorectal lesions containing Russell bodies are extremely rare or are probably underreported with three case reports published in the English literature in a PubMed search from 1980 to date [4–6]. One of these three cases presented as a Colonic Russell body inflammatory polyp [4]. This review discusses the clinical and endoscopic features, histopathologic features, ancillary studies, pathogenesis, differential diagnosis, prognosis, and treatment of this rare entity.

## 2. Clinical and Endoscopic Features

Colorectal Russell body lesions are extremely rare. The patient characteristics and clinical and endoscopic features of the 3 cases of colorectal Russell body lesions reported in the literature to date are tabulated in Table 1. Of note, two of these presented as colorectal polyps (one patient with Colonic Russell body inflammatory polyp and the other one as tubulovillous adenoma with high-grade dysplasia with dense lamina propria plasma cell infiltrates containing Russell bodies and Mott cells), while one showed normal colonoscopy [4–6]. The only patient of Colonic Russell body inflammatory polyp (Table 1, case 1) reported in the literature had associated severe diverticulosis in the transverse colon, splenic flexure, descending colon, and sigmoid colon [4]. The patient with normal colonoscopy (Table 1, case 2) showed diffuse involvement by Russell bodies, associated with an immunocompromised state (status post kidney and pancreas transplant).

Though a common occurrence in the hematopathology specimens, Russell body containing lesions are unusual in the gastrointestinal (GI) tract with approximately 40 GI cases reported in the literature to date. In the GI tract, they are most commonly seen in the stomach as Russell body gastritis and have been associated with* Helicobacter pylori* infection in some studies; however this association has not been definitively proven [7–16]. Other cases have been reported as Russell body duodenitis and Russell body esophagitis [7, 17–20]. The Russell body containing lesions in the GI tract span a wide age spectrum ranging from 24 to 88 years of age with a reported male to female ratio of 1.6:1 [5]. The clinical presentation of the upper GI Russell body containing lesions is nonspecific and varies from abdominal pain, nausea, dyspepsia, and diarrhea. Endoscopic features in the upper GI tract are again nonspecific and consist of mucosal erythematous changes, erosion/ulcers, edema, or rarely nodules [5].

## 3. Histopathologic Features

**Figure 1:**
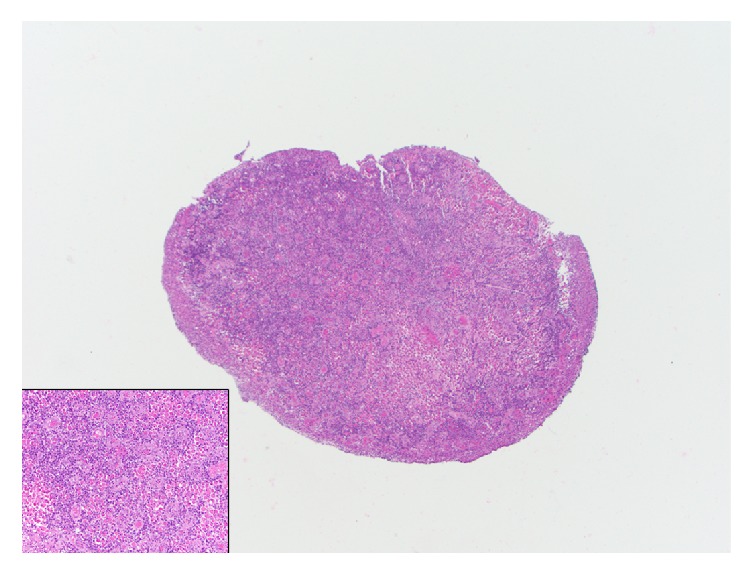
Low power photomicrograph showing a Russell body inflammatory polyp with associated surface ulceration and granulation tissue. Inset shows a high power photomicrograph of colorectal Russell body containing lesion highlighting the Mott cells, which are plasma cells containing eosinophilic globules (H&E X40, inset H&E X400).

**Figure 2:**
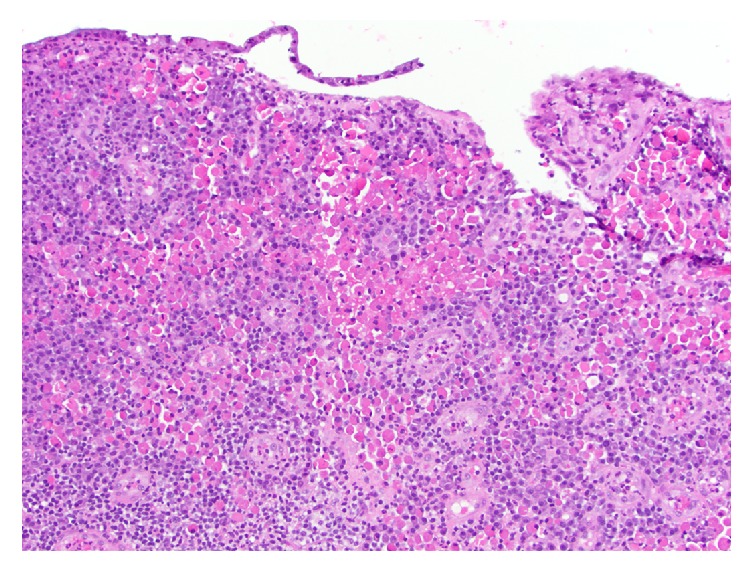
Medium power photomicrograph highlighting Russell body inflammatory polyp with surface epithelial erosion and expansion of lamina propria by Mott cells as well as other inflammatory cells (H&E X 200).

Histologically, Russell body inflammatory polyp typically exhibits features of inflammatory polyp with expansion of lamina propria by Mott cells. The surface epithelium can be intact and show hyperplastic changes or can be partially or totally eroded with the presence of ulcer and granulation tissue (Figures 1 and 2). The lamina propria in the colorectal Russell body lesions (including Russell body inflammatory polyp) shows expansion by inflammatory cells including variable composition of neutrophils, plasma cells, lymphocytes, and eosinophils. However, the most prominent population is of Mott cells, which are plasma cells showing eccentric nuclei and containing numerous intracytoplasmic eosinophilic globules also known as Russell bodies (Figure 1 inset) [3].

The histopathologic features of the 3 cases of colorectal Russell body containing lesions are tabulated in Table 1.

## 4. Ancillary Studies

**Figure 3:**
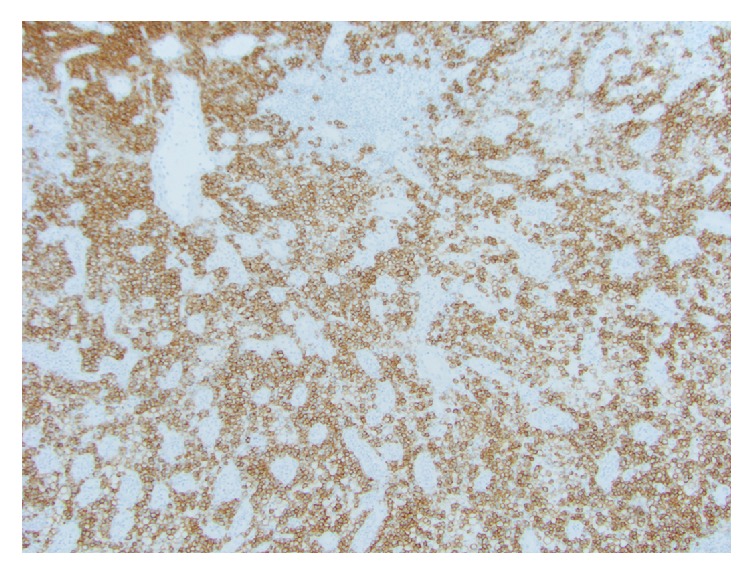
CD138 immunohistochemical stain highlights the Mott cells in colorectal Russell body containing lesion (CD138 X 100).

**Figure 4:**
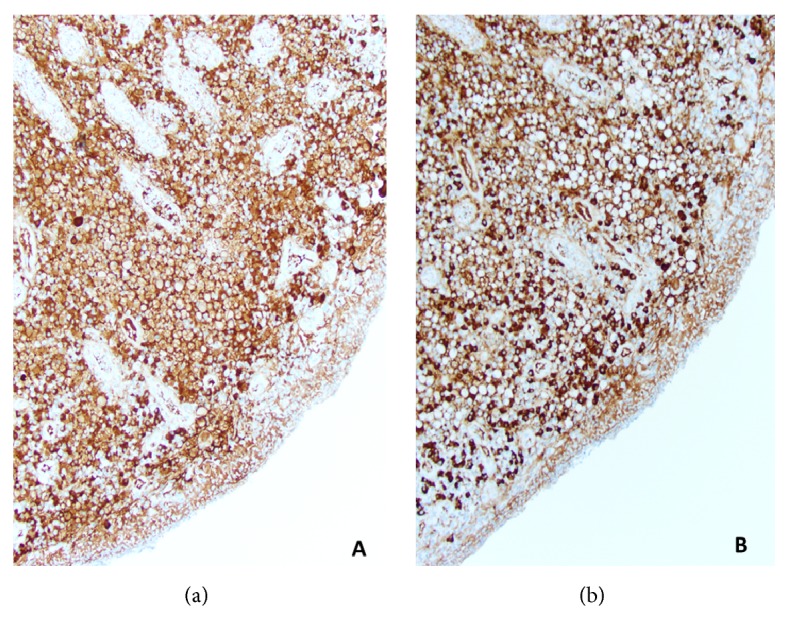
Polyclonal plasma cells seen in Russell body inflammatory polyp as demonstrated by Kappa (a) and lambda (b) light chain expression (Kappa, Lambda x200).

The Russell bodies are periodic acid-Schiff positive. By immunohistochemistry, the Mott cells in Russell body inflammatory polyps are diffusely positive for plasma cell markers, CD138 (Figure 3) and CD79a, and are negative for pancytokeratins. Typically, the plasma cells are polyclonal as demonstrated by immunohistochemistry and/or in situ hybridization, for Kappa or Lambda light chain expression (Figures 4(a) and 4(b)). However, certain cases of monoclonality in GI tract Russell body containing lesions have been reported, without evidence of a neoplastic lesion [4–9, 20].

The ancillary studies in reported cases of colorectal Russell body containing lesions are tabulated in Table 1.

## 5. Pathogenesis

Russell body containing lesions in the colorectum is considered a rare vigorous form of inflammatory response to chronic mucosal inflammation, possibly leading to overstimulation of plasma cells and accumulation of numerous nondegradable immunoglobulins in cisternae of rough endoplasmic reticulum. During episodes of chronic inflammation, Mott cells can present as individual cells or clusters or sheets of cells in the lamina propria or accumulate to form aggregates known as Russell body inflammatory polyp, as seen in the patient of severe diverticulosis presenting with Russell body inflammatory polyp [4, 6]. However, why this response is seen in some subset of patients with diverticulosis or chronic inflammation and not all remains uncertain. It is also possible that these cases may be underreported in the literature.

Overall, majority of cases of Russell body gastroenteritis have been reported in upper GI tract, predominantly in the stomach, and have been associated with* Helicobacter pylori* infection in multiple studies [7–14]. Although Russell body proliferation is a benign entity, it has rarely been reported in association with gastric malignant epithelial neoplasms such as signet ring cell carcinoma, Epstein-Barr virus-associated gastric carcinoma, and gastric tubular adenocarcinoma [7, 21–23].

## 6. Differential Diagnosis

Though considered benign, the importance of Russell body inflammatory polyp or Russell body containing lesion lies in recognizing this unusual entity and differentiating it from its malignant mimics such as a signet ring cell carcinoma, which comes rarely in the differential [24, 25]. Unlike the former, the latter shows nuclear atypia and is positive for cytokeratin and mucicarmine. Russell body containing lesion lacks nuclear atypia, is negative for cytokeratins, and is positive for plasma cell markers. The other differential includes plasma cell neoplasms, such as mucosa associated lymphoid tissue (MALT) lymphoma with plasmacytic differentiation and plasmacytomas, and these typically exhibit cellular atypia and mitotic activity and are monoclonal, unlike most cases of Russell body containing lesions.

## 7. Prognosis and Treatment

Logically, Russell body inflammatory polyp or lesions can be treated by polypectomy as well as by treatment or reduction of the underlying cause of chronic inflammation. Russell body polyps of the upper GI tract have been seen in association with* H. pylori* gastritis, and some studies have shown that Russell body polyps occurring in the setting of* H. pylori* will be eliminated by the eradication of the concurrent infection [8–14].

## 8. Conclusions

Russell body inflammatory polyp and Russell body lesion with Mott cells are exceedingly rare in the colorectum and have only been described in the literature in the form of three case reports from 1980 to date. These are benign, reactive lesions most likely secondary to overproduction of immunoglobulins and development of Mott cells with Russell bodies as a response to chronic mucosal inflammation. The clinical significance lies in recognizing the benign and reactive nature of this entity, assessing the causative etiology, and differentiating it from its malignant mimics which come in the histologic differential.

## Figures and Tables

**Table 1:** Case reports of Russell body containing Mott cells in the colorectum.

Case no. (Source)	Age, year/ Sex	Location	Clinical Presentation	Endoscopic findings	Histopathologic findings	Ancillary Studies
1 (Coates et al., 2017 [4])	62/Male	Sigmoid Colon	Average risk screening colonoscopy, no GI complaints	<5 mm Erythematous polyp; severe diverticulosis	Inflammatory polyp with Russell bodies in the lamina propria.	CD138 and CD79a positive; *∗*polyclonal

2. (Muthukumarana et al., 2015 [5])	44/Female	Colon	Diarrhea, abdominal pain, nausea and vomiting. Status post kidney and pancreas transplant with immunosuppression.	Normal colonic endoscopy; no colonic polyp	Chronic lymphoplasmacytic infiltrate with Mott cells within the lamina propria in colon, terminal ileum, duodenum and stomach	PAS positive; CD138 positive; *∗*polyclonal

3 (Brink et al., 1999 [6])	53/Female	Rectum	Indication not provided. No evidence for malignant myeloma or a gammopathy.	Rectal polyp, not further specified	Tubulovillous adenoma with high-grade epithelial dysplasia and dense plasma cell infiltrates containing Russell bodies and Mott cells	IgG monoclonal for kappa light chain

*∗* polyclonal nature confirmed by concomitant expression of kappa and lambda light chain.
